# The Passing of Parasyphilis

**Published:** 1914-05-09

**Authors:** 


### RESEARCH AND REMEDIES.

### The Passing of Parasyphilis.

Under this dashing headline Pollitzer discusses in the *Medical Record* the effect of recent work by Noguchi upon the current views concerning tabes and general paralysis, and their relations to syphilis. He points out that it is only in the last sixty years that tabes has been recognised as a disease almost invariably preceded by syphilis, and about half that time since the same became generally admitted concerning general paralysis. Yet anti-syphilitic treatment, such as sufficed to clear up other syphilitic manifestations, failed to benefit either of these two conditions. To account for this it was supposed, and is still very generally believed, that although these diseases occur almost exclusively in those who have had syphilis, and depend on a previous syphilitic infection, yet they are not actually manifestations of syphilis. In accordance with this idea they have been dubbed "parasyphilitic" diseases. Now, however, Noguchi claims to have demonstrated spirochætes in the brain in forty-eight out of two hundred cases of general paralysis. They are sparse and difficult to find; but it seems likely that improved technique may prove them to be present in every case. Thus "parasyphilitic" will be relegated to the limbo of forgotten catchwords; and paresis and tabes will rank as forms of cerebral and spinal syphilis, with correspondingly enhanced hope of cure.

